# Maternal Serum Disintegrin and Metalloprotease Protein-12 in Early Pregnancy as a Potential Marker of Adverse Pregnancy Outcomes

**DOI:** 10.1371/journal.pone.0097284

**Published:** 2014-05-15

**Authors:** Jiexia Yang, Jing Wu, Fangfang Guo, Dongmei Wang, Keyi Chen, Jie Li, Li Du, Aihua Yin

**Affiliations:** 1 Prenatal Diagnosis Centre, Guangdong Women and Children Hospital, Guangzhou, Guangdong, China; 2 Maternal and Children Metabolic-Genetic Key Laboratory, Guangdong Women and Children Hospital, Guangzhou, Guangdong, China; Bascom Palmer Eye Institute, University of Miami School of Medicine, United States of America

## Abstract

**Objectives:**

The aim of this study was to determine whether the concentration of disintegrin and metalloprotease protein12 (ADAM12) in first trimester maternal serum can be used as a marker for first-trimester complete spontaneous abortions, missed abortions, ectopic pregnancies and hydatidiform moles.

**Methods:**

The maternal serum concentrations of ADAM12 were measured in the range of 5–9^+6^ weeks of gestation using an automated AutoDelfia immunoassay platform in 9 cases of complete spontaneous abortion, 27 cases of missed abortions, 56 cases of ectopic pregnancies, 12 cases of hydatidiform moles, and 100 controls. Logistic regression analysis was used to determine significant factors for predicting adverse pregnancy outcomes in early pregnancy. Screening performance was assessed using receiver operating characteristic curves.

**Results:**

Two hundred and four women were enrolled in the study. In the control group, the level of ADAM12 increased with gestational age. The median ADAM12 levels in the spontaneous abortion (0.430 MoM), ectopic pregnancy (0.460 MoM) and hydatidiform mole (0.037 MoM) groups were lower than that in the control group, while the median ADAM12 level in the missed abortion group (1.062 MoM) was not significant from the controls (1.002 MoM). Logistic regression analysis demonstrated that the level of ADAM12 in maternal serum facilitated the detection of ectopic pregnancies (OR = 0.909; 95% CI = 0.841∼0.982) and complete spontaneous abortion (OR = 0.863; 95% CI = 0.787∼0.946).

**Conclusions:**

In complete spontaneous abortion and ectopic pregnancy, ADAM12 maintained at low levels in early pregnancies, and there were significant differences compared to normal pregnancies. ADAM12 is a promising marker for the diagnosis of complete spontaneous abortion and ectopic pregnancy in symptomatic women, and under certain conditions, ADAM12 can diagnose ectopic pregnancy and spontaneous abortion before an ultrasonographic detection of the conditions.

## Introduction

With the social and natural environment deteriorating, the incidence of adverse pregnancy outcome showed amplification trends, and the chance of having a healthy baby reduce. Miscarriage in the first trimester affects approximately 15% of all pregnancies. [1] Miscarriage includes complete spontaneous abortion and missed abortion. Ectopic Pregnancies (EP) is a major cause of maternal morbidity and remains the most life-threatening acute condition in modern gynecology in early pregnancy. The diagnosis of EP remains a major clinical challenge in obstetrics, with patients often being asymptomatic or presenting with non-specific symptoms that do not differentiate EPs from normal pregnancies. The ability to identify factors early in pregnancies to predict women with higher risk of developing adverse pregnancy outcomes would be helpful in planning prenatal care.

A disintegrin and metalloprotease12 (ADAM12), a multi-domain glycoprotein belong to the reprolysin zinc metalloprotease family [2], [3] ectopic pregnancies, highly expressed in placental tissue [4], was supposed to be involved in controlling placental and fetal growth and development. As a marker for adverse pregnancy outcomes in early pregnancy, ADAM12 has been shown to be lower in maternal serum,,subsequently developed into an EP [5]. However, a recent study reported that serum ADAM12 concentrations in a UK cohort elevated in women with histologically-confirmed EPs compared with women with viable intrauterine pregnancies (VIUPs) [6]. In addition, ADAM12 levels have been shown to decrease in pregnancies that progress to complete spontaneous abortion. [7] To date, no much published studies have investigated ADAM12 levels and first trimester adverse pregnancy outcomes, particularly including complete spontaneous abortions, missed abortions, EPs, and hydatidiform moles. In the current study, we evaluated whether maternal ADAM12 levels in the first trimester could be used to predict subsequent adverse pregnancy outcomes.

## Materials and Methods

A total of 204 unrelated gravidas, including women with adverse and normal pregnancy outcomes, were enrolled in the study under an approved protocol of informed consent by the Guangdong Women and Children Hospital Institutional Review Board and Ethics Committee of Guangzhou Medical College (China). Each participant signed a written informed consent, which guaranteed that every specimen was used for scientific research only, and not used for commercial or other purposes. All informed consents were organized into files and archived. Informed consent procedure was approved and monitored by the Ethics Committee.

The aim of this case-control study was to identify the differences between women who experienced a normal pregnancy (control) and those who experienced adverse pregnancy outcomes (cases) being treated at the Prenatal Diagnosis Center of Guangdong Women and Children’s Hospital between June 2010 and July 2011. Gestational age was determined from the known date of the last menstrual period and recalculated by ultrasonographic measurement of the crown-rump length when exceeding > a 7 day difference from the last menstrual period. Selecting gestation in 5–9^+6^ weeks of all pregnant women, under the condition of informed consent detect pregnant women ADAM12 concentration in peripheral blood and follow-up regularly for all Pregnant woman, Excluding twins and multiple pregnancies based on the results of follow-up then grouping, All adverse pregnancy outcomes are diagnosed by ultrasound.

Approximately 5 ml of peripheral blood were collected from patients, and left to clot at room temperature for 30 min. The blood samples were then centrifuged at 3,000 g for 10 min and serums were collected and stored at −70°C until assayed. The incidences of complete spontaneous abortion, missed abortion, EP and hydatidiform mole during 5–9^+6^ weeks gestational age were evaluated. A complete abortion means that the body has expelled all the products of pregnancy (blood, tissue, embryo) and there is no need for surgery (vacuum aspiration) afterwards. Missed abortion is a pregnancy in which there is a fetal death without outside intervention, also called a silent miscarriage, can happen in any pregnancy, embryonic can not natural discharge after stopping growth. An EP is an abnormal pregnancy that occurs outside the uterus. The final diagnosis was confirmed by ultrasound. Hydatidiform moles are abnormal pregnancies characterized histologically by aberrant changes within the placenta. Classically, the chorionic villi in these placenta show varying degrees of trophoblastic proliferation and edema of the villous stroma.

In the current study, we measured maternal ADAM12 in 215 cases, of which 11 were excluded since they rejected follow-ups. The remaining cases include 9 of complete spontaneous abortions, 27 of missed abortions, 56 of EPs, 12 of hydatidiform moles and 100 controls. The samples were used to measure ADAM12 concentrations using a time-resolved fluorescent immunoassay with an AutoDelfia ADAM12 platform (PerkinElmer Life and Analytical Sciences, Turku, Finland).

Statistical analysis was performed with SPSS (version 13 for Windows; SPSS, Inc., Chicago, IL, USA) and data were reported as the mean±standard deviation for continuous variables that were normally distributed and as the median and interquartile range (IQR) for non-normally distributed variables. The Kruskal-Wallis test was used to determine the significance of differences in the median ADAM12 level for each adverse outcome group to the control group. Logistic regression analysis was used to determine which of the factors among the maternal characteristics ADAM12 had a significant contribution in predicting adverse pregnancy outcome. The performance of screening was determined by receiver operating characteristic (ROC) curves.

## Results

204 women were enrolled in the study. The maternal characteristics of each of the outcome groups are shown in **Table 1**. The median maternal age in the study population was 27.5 years (range, 19–40 years). The median gestational age was 6 weeks (range, 5–9^+6^weeks).

**Table 1:** Maternal characteristics of the five outcome groups.

Outcome group	Maternal age (years)	Gestational age (weeks)
Control (n = 100)	27 (22–38)	6 (5–9)
complete spontaneous abortion (n = 9)	26 (19–40)	7 (5–8)
Missed abortion (n = 27)	29.5 (23–37)	7 (5–9)
Ectopic pregnancy (n = 56)	27.4 (23–38)	6 (5–9)
Hydatidiform mole (n = 12)	28 (20–36)	7.5 (5–10)

Note: Data are the median (range).

Statistical tests of significance were by comparison with the control group using the Kruskal-Wallis test.

**Figure 1:**
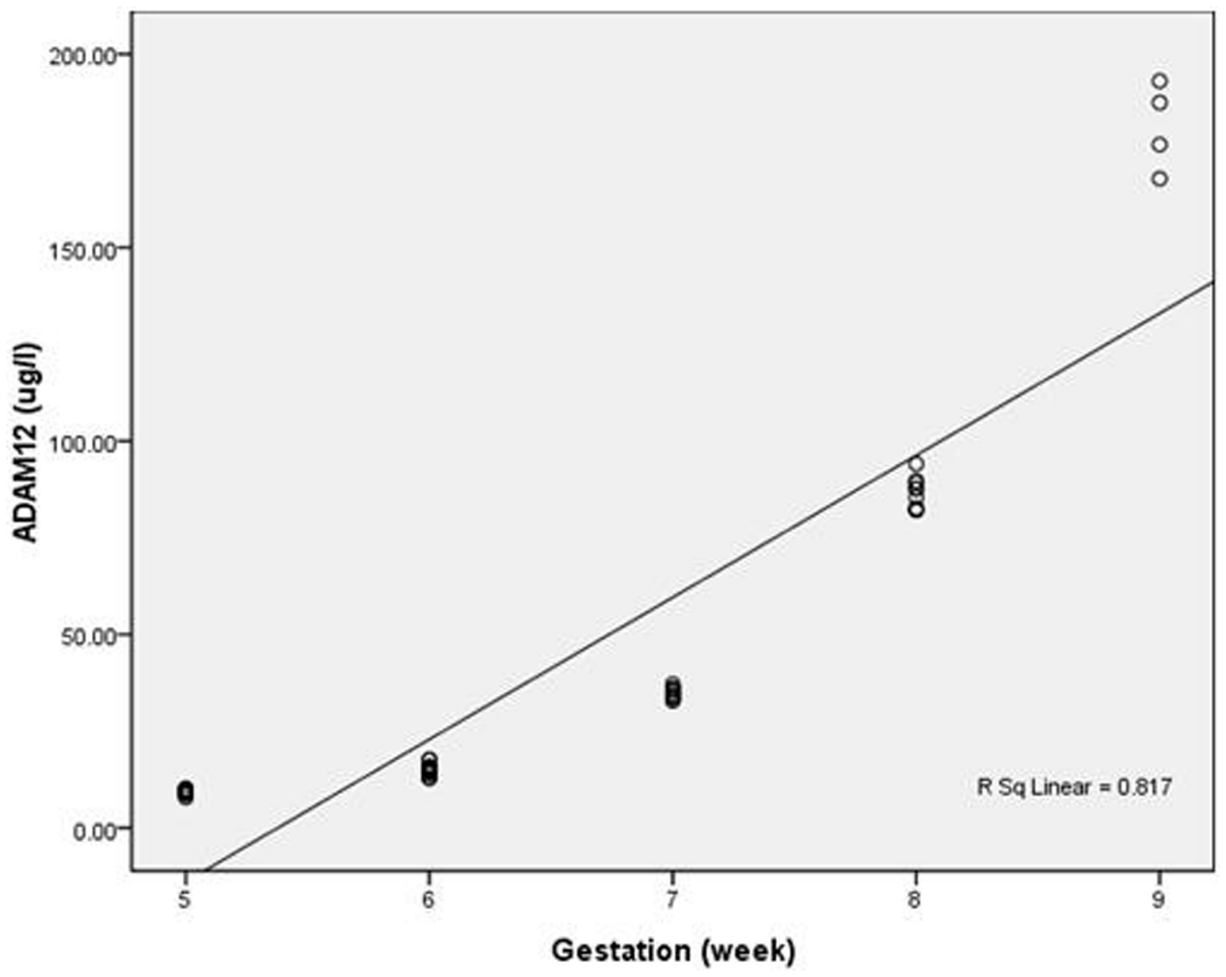
Variation of maternal serum ADAM-12 concentration with gestational age in the control group Curve.

In the control group, the concentration of ADAM12 increased with gestational age from 5 to 9^+6^ weeks (**Figure 1**). The different gestational maternal serum ADAM12 MoMs are shown in **Table 2** **.**

**Table 2:** Maternal serum ADAM12 MOM with different gestational age of the control group.

Gestational age (weeks)	Numbers(n)	ADAM12- MOM
5∼	15	0.976±0.079
6∼	20	1.003±0.092
7∼	24	1.000±0.042
8∼	24	0.990±0.046
9∼	17	0.996±0.062

In the complete spontaneous abortion group, compared with the control group, the median ADAM12 concentration level was lower. In the missed abortion group, there was no significant difference in the concentration of ADAM12 compared with the control group. In the EP and the hydatidiform mole groups, ADAM12 levels were lower than the control group. The results are shown in **Table 3**.

**Table 3:** Comparison of the median level of ADAM12 in each adverse outcome group with the control group.

Outcome group	MOM	Interquartile range
Control	1.002	0.941∼1.032
complete spontaneous abortion	0.430	0.172∼0.760*
Missed abortion	1.062	0.268∼2.383
Ectopic pregnancy	0.460	0.077∼0.634*
Hydatidiform mole	0.037	0.005∼0.348*

ADAM12: a disintegrin and metalloprotease-12.

*P<0.05.

Logistic regression analysis was used to determine the association between the concentration of ADAM12 and the risk of adverse pregnancy outcomes. After adjusting for gestational and maternal ages, logistic regression analysis demonstrated that the ADAM12 level could facilitate the detection of adverse outcomes (**Table 4**) except in the missed abortion group, the difference of ADAM12 level was not statistically significant.

**Table 4:** Logistic regression analysis for the prediction of adverse outcome groups.

Outcome group	OR	95% CI	*P*
complete spontaneous abortion	0.863	0.787∼0.946	0.002
Missed abortion	1.003	0.997∼1.008	0.358
Ectopic pregnancy	0.909	0.841∼0.982	0.015
Hydatidiform mole	0.509	0.318∼0.813	0.005

OR, odds ratio; CI, confidence interval; ADAM12, a disintegrin and metalloprotease-12.

**Figure 2:**
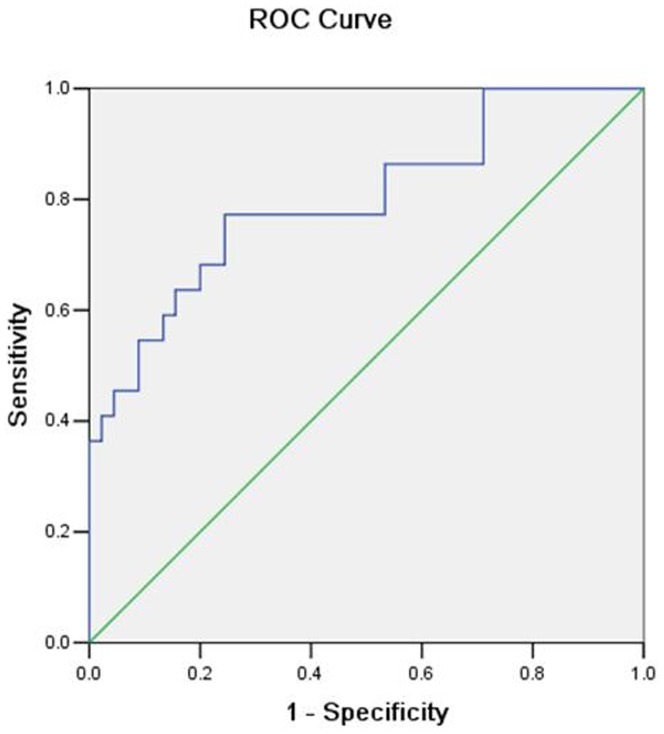
Receiver-operating characteristic (ROC) plots for ADAM-12 in the prediction of ectopic pregnancy.

**Figure 3:**
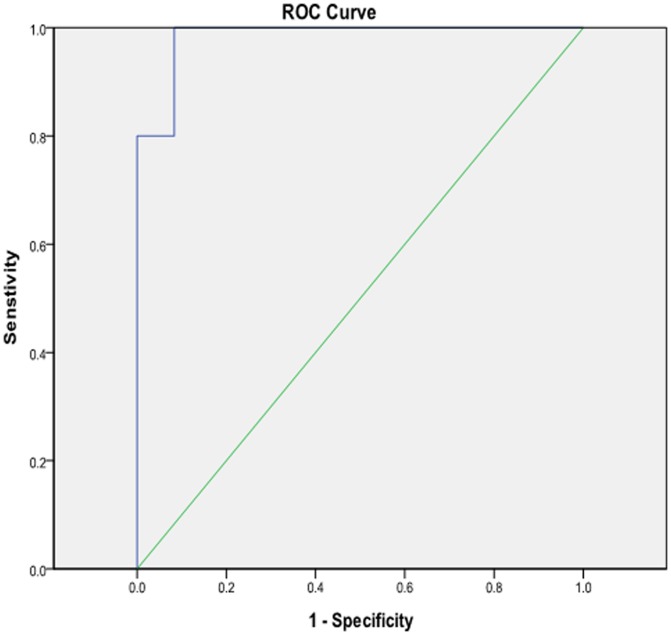
Receiver-operating characteristic (ROC) plots for ADAM-12 in the prediction of hydatidiform mole.

**Figure 4:**
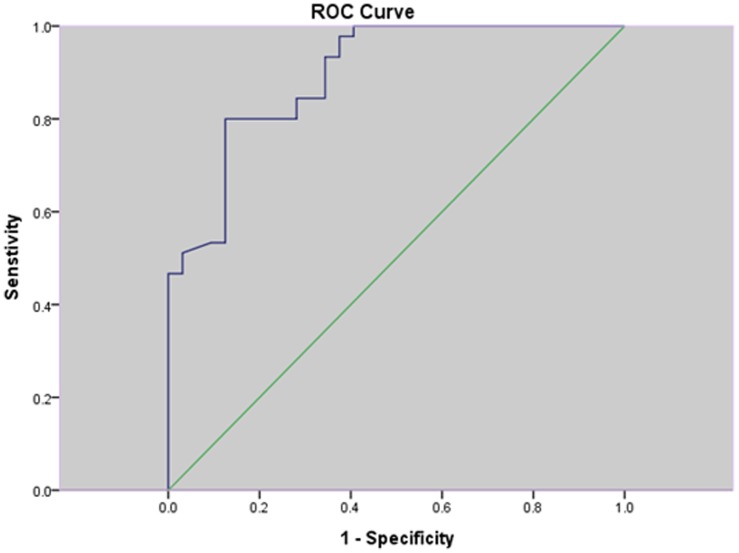
Receiver-operating characteristic (ROC) plots for ADAM-12 in the prediction of complete spontaneous abortion.

ADAM12 predicting with adverse pregnancy outcomes that is confirmed by the ROC curves shown in Figures(**Figures 2** **-** **4**). The area under the curve (AUC) was 0.80. Using the maternal serum ADAM12 level 13.84 ug/l as a cut-off value to predict EP (**Figure 2**), the sensitivity was 77.3% and the false-positive rate was 24.4%. The area under the curve (AUC) was 0.98. Using the maternal serum ADAM12 level 5.45 ug/l as a cut-off value to predict hydatidiform mole (**Figure 3**), the sensitivity was 100.0% and the false-positive rate was 8.3%; the area under the curve (AUC) was 0.90. Using the maternal serum ADAM12 level 12.85 ug/l as a cut-off value to predict complete spontaneous abortion (**Figure 4**), the sensitivity was 80.0% and the false-positive rate was 12.5%.

## Discussion

The findings of the current study demonstrate that at 5−9^+6^ weeks gestation, in the complete spontaneous abortion, ectopic pregnancy and hydatidiform mole groups, ADAM12 levels are lower than that in the control group. The MOM ADAM12 levels of the complete spontaneous abortion, ectopic pregnancy and hydatidiform mole groups are lower than the control group. The ADAM12 level in the missed abortion group is not significantly different from that in the controls. The data show that ADAM12 can discriminate the complete spontaneous abortion, the EP and the hydatidiform mole from normal pregnancies with good sensitivity and specificity, and suggest that the value of ADAM12 can be used as a potential biomarker for adverse pregnancy outcomes. Besides, maternal factors contribute significantly to the prediction of adverse pregnancy outcomes, especially gestational age.

DAM12 may has some potential as a serum biomarker for complete spontaneous abortion. Wu et al. [7] reported that the measurement of ADAM12 in pregnant women has high accuracy in the prediction of spontaneous abortion; In the present study, we demonstrated a difference in the serum ADAM12 level between the complete spontaneous abortion group and the normal pregnancy group. The MoM of ADAMl2 in complete spontaneous abortion was 0.430 (0.172∼0.760*)and the control group 1.002 (0.941∼1.032), with significant difference. Considering the small sample size in the current study, further researches are needed to confirm whether ADAM12 is reliable in predicting complete spontaneous abortion.

ADAM12 has been studied as a first trimester marker for predicting pre-eclampsia [8]–[10], aneuploidy [8], [10]–[15] and small-for-gestational age fetuses [16], [17]. A clear biological explanation for the decreased level of ADAM12 in EPs does not exist; however, the predominant theory involving impaired placentation could account for ADAM12 playing an important role in the growth and function of the placenta [8]. ADAM12 is produced by the placenta [18] and is present in the serum of gravidas, but not in the serum of non-pregnant women. [4] If ADAM12 is involved in the normal implantation of pregnancy, and decreased levels reflect abnormal implantation or serve as a harbinger of an abnormal pregnancy, then decreased levels of ADAM12 in EP may be biologically plausible.

Prior studies have demonstrated that maternal serum ADAM12 increases with gestational age, [10], [13], [19] but there is a paucity of data involving normal pregnancies prior to 7 weeks gestation. The results of the current study showed that the maternal serum ADAM12 concentration in normal pregnancies increased with gestational age between 5 and 9 weeks gestation. Sahraravand et al [20] reported that gestational age is likely to be a key factor in ADAM12 levels, as ADAM12 rises exponentially from approximately week 5 of the first trimester.

With the social environment and people's living habits change, the frequency of adverse pregnancy outcomes showed expansion trend. In severe cases, it is even life-threatening, thus early discovery and early diagnosis is essential. ADAM12 can diagnose ectopic pregnancy and spontaneous abortion before the ultrasonographic detection of the conditions, and can be used as dynamic monitoring of early pregnancy, If adding progesterone and human chorionic gonadotropin comprehensive judgment detection rate is higher.

It is important to note that the sample size in the current study was relatively small. Thus, the findings should be confirmed in large sample studies, from which it would be possible to estimate the screening performance of ADAM12. Nevertheless, the finding represented an important step towards establishing a method for screening EP, complete spontaneous abortion and hydatidiform mole in the first trimester. If further studies can confirm the reduction of ADAM12 in the first trimester, a strategy including ADAM12 may be adopt to identify high-risk women and to prevent untoward outcomes.
